# Just Say No to the TPP: A Democratic Setback for American and Asian Public Health: Comment on "The Trans-Pacific Partnership: Is It Everything We Feared for Health?"

**DOI:** 10.15171/ijhpm.2016.145

**Published:** 2016-11-06

**Authors:** Carles Muntaner, Deb Finn Mahabir

**Affiliations:** ^1^Bloomberg Faculty of Nursing University of Toronto, Toronto, ON, Canada.; ^2^Dalla Lana School of Public Health, University of Toronto, Toronto, ON, Canada.; ^3^School of Medicine, University of Toronto, Toronto, ON, Canada.

**Keywords:** Scientific Realism, Health Policy, Politics, Epistemology, Causality, Social Mechanisms

## Abstract

The article by Labonté, Schram, and Ruckert is a significant and timely analysis of the Trans-Pacific Partnership (TPP) policy and the severe threats to public health that it implies for 12 Pacific Rim populations from the Americas and Asia (Australia, Brunei, Canada, Chile, Japan, Malaysia, Mexico, New Zealand, Peru, Singapore, United States, and Vietnam). With careful and analytic precision the authors convincingly unearth many aspects of this piece of legislation that undermine the public health achievements of most countries involved in the TTP. Our comments complement their policy analysis with the aim of providing a positive heuristic tool to assist in the understanding of the TPP, and other upcoming treaties like the even more encompassing Transatlantic Trade and Investment Partnership (TTIP), and in so doing motivate the public health community to oppose the implementation of the relevant provisions of the agreements. The aims of this commentary on the study of Labonté et al are to show that an understanding of the health effects of the TPP is incomplete without a political analysis of policy formation, and that realist methods can be useful to uncover the mechanisms underlying TPP’s political and policy processes.

The article by Labonté, Schram, and Ruckert^1^ (this issue), henceforth LSR, is a significant and timely analysis of the Trans-Pacific Partnership (TPP) policy and the severe threats to public health that it implies for twelve Pacific Rim populations from the Americas and Asia (Australia, Brunei, Canada, Chile, Japan, Malaysia, Mexico, New Zealand, Peru, Singapore, United States, and Vietnam). With careful and analytic precision the authors convincingly unearth many aspects of this piece of legislation that undermine the public health achievements of most countries involved in the TTP. Our comments will complement their policy analysis with the aim of providing a positive heuristic tool to assist in the understanding of the TPP, and other upcoming treaties like the even more encompassing Transatlantic Trade and Investment Partnership (TTIP), and in so doing motivate the public health community to oppose the implementation of the relevant provisions of the agreements. The aims of this commentary on LSL’s study are twofold: to argue that an analysis of the health effects of the TPP is incomplete without a political analysis of its policy formation^2,3^ and that realist methods can be useful to uncover the mechanisms underlying TPP’s political and policy processes.^4^

LSR start their policy analysis of the TPP with the agreed upon policy document. Yet, the policy process and its underlying political struggles that lead to this multilateral agreement are crucial to understanding its putative public health consequences, including whether the policy should be implemented or not.^5^ Such a task might require new methods^6^ (see section below). In the meantime, our preliminary analysis of actors and strategies during the TPP’s agenda setting and policy formulation^7^ processes reveals the central role that political power plays in the agreement.

The agenda setting of the TPP agreement (or those of the TTIP, World Trade Organization (WTO), North American Free Trade Agreement [NAFTA], Central America Free Trade Agreement [CAFTA], and US treaties with China and South Korea) are built around the assumption that “free trade” is a beneficial “win-win” strategy for the countries involved in terms of economic development and quality of life for their population.^8^ Yet, the evidence from economic history for example, points to major development successes fueled by trade protectionism.^8-10^ Indeed, trade protectionism has enabled social and health progress in countries like South Korea, Taiwan, and Japan.^8-10^ Conversely, trade liberalization has been associated with health hazards such as increased consumption of tobacco, alcohol, and foods high in salt, fat, and sugar.^11^ In spite of talk about free trade the TPP is not primarily about reducing tariffs, as one would expect, since they are already low^5,12-15^ but rather about investor’s rights, including the right to challenge democratically elected governments in regulations that protect the health of populations via the investor-state dispute settlement (ISDS).^5,12,15^ In its essence, the TPP is a way of “harmonizing down” to the benefit of the private sector over and above democratically sanctioned regulations that protect the health of the public.^12-16^ A policy formation^7^ analysis is needed to understand the secrecy of the meetings behind the publics’ back, without civil society participation, yet with hundreds of lawyers and lobbyists representing corporate interests devoted to scrutinizing every single page of the agreement.^12,13^

Simply stated, these strategies reveal an unfair power imbalance in the TPP’s policy formation stage. First, the TPP serves to get rid of (democratically elected) government regulations that stand in the way of corporate profits.^12,15^ Second, the TPP undermines countries in their ability to challenge large multinational corporations. For example, using the ISDS panels (whose staff are selected by corporations), a tobacco company could sue a country if its tobacco regulations would threaten its profitability prospects. While in wealthy countries such as Australia, Canada, and the United Kingdom, states have won trials against tobacco companies,^16^ the TPP’s ISDS would substantially shift the power balance in favor of corporations.^12,13,15^

In examining the LSR adaptation of the health impact assessment (HIA) approach, several methodological innovations could have contributed to a better understanding of the health implications of the TPP.^17-24^ Although, a HIA analysis is typically conducted using quantitative methods (a reproducible transparent method), at issue is that in order to achieve a deeper political and policy analysis of the policy formation process of the TPP (and other similar trade agreements) we need qualitative methods that remain reproducible and transparent.^4,25,26^ This means that an adaptation of the HIA to a qualitative approach requires that, several core tenets (to be discussed in later paragraphs) must be observed in order to maintain the reproducibility (and thus validity) of findings.

The current adaption of the HIA tool in LSL amounts to a pragmatic approach which is useful yet has some limitations with regard to explaining the TPP. Specifically, the knowledge derived from LSL’s textual analysis is developed without a model, hypotheses or explicit procedure to test them. Such an approach, thus, limits the ability to systematically identify social causal mechanisms (eg, a common activity of policy actors involved in the TPP is to rewrite the rules of the economy that can undermine democratically established government regulations).^15^ This lack of social causal mechanisms reduces the potential for recommendations leading to policy interventions^18-27^ (eg, reforms to protect democratic government regulations from the interference of private actors in trade agreements). In brief, a mere reading of the TPP final draft without political theory does not permit one to systematically uncover the underlying explanatory social causal mechanisms at play during its policy formation stages. Furthermore, the pragmatic approach used in LSL’s analysis is less heuristic than a realist approach,^4,5,25,26^ since it inhibits the development and testing of hypothesis from which to then refine theory or draw policy recommendations.

We maintain that a systematic theory-based analysis^4^ of policy formation^7^ and a testing of hypothesis or models, is needed to reveal the distribution of power relations in the crafting of the TPP and its important consequences for the social determinants of health (SDOH). The approach used in LSL’s paper to understand the TPP’s consequences for population health, limits the analysis to the examination of the text of the agreement. We should avoid this danger of “textualism” whereby the analysis of society is limited to a textual analysis.^17^ For example, this approach to the TPP fails to capture political processes that maintain the status quo for the wealthy. Based on the experience from previous trade agreements,^13^ the TPP most likely will result in an increase in economic inequality since its gains will only benefit the wealthiest.^5,12-15^ In addition, environmental, food, occupational, and healthcare regulations could be challenged if they are perceived as a threat to profitability via the ISDS instrument, whose composition is predisposed towards investor interests.^5,12,15^ Essentially, there is potential in the TPP for negatively impacting the SDOH.

We propose a set of alternative methods.^4,6,18-27^ Specifically, we assert that a scientific realist (SR) approach^20^ offers several critical advantages that need to be considered to effectively analysis the HIA using qualitative methods. Epistemologically, a SR analysis asserts that an objective truth exists and, thus, a real world exists that is independent of the observer. Moreover, this reality is stratified into the empirical (experienced and perceived), the actual (events and outcomes occur but may not be perceived), and the real (where underlying structures as emergent properties and mechanisms can cause changes and outcomes).^18^ Additionally, real objects are considered intransitive, which means that these objects exist independent of our knowledge or perception of them.^22^ In other words, this SR approach seeks to identify causal social mechanisms that are hidden, such as power relations. Therefore, a SR approach supports our understanding of society by making the “black box” of policy formulation transparent through the identification of causal mechanisms that are linked to specific contexts.^23^ This search for causal mechanisms in turn supports the needed explanation of how, why, for whom, and under what circumstances specific outcomes occur,^6^ such as how and why the power of private actors influence the policy process and policy outcomes of HIA.

This realist epistemology is currently adopted in the social sciences^24^ and public health.^18^ For example, realist methods have been previously used to systematically reveal social mechanisms involved in the implementation of Health in all Polices (HiAP).^4,25^ In using a social conflict theoretical approach, the evidence from these studies demonstrates that outcomes of HiAP policy implementation are linked to hypotheses and social mechanisms involving ideology, resistance, and political power, something that would be important to uncover in a critique of the TPP.

In terms of transparency and reproducibility, realist methods start with a theory from which testable hypothesis are developed with the aim of re-fining a specific theory. Next, the search for mechanisms using realist methods are used for theory testing in order to advance the understanding of policy formation, implementation, or evaluation.^18,26,27^ Realist methods use data from multiples sources (for example, from key informant interviews, grey and empirical literature) to increase validity. Ultimately, these processes specific to a SR analysis could provide trade agreement scholars with a transparent and reproducible method. Moreover, the SR approach provides a methodology to systematically identify mechanisms during the process of policy formulation which can inform a critical analysis of public polices and interventions. The realist approach can also be used in conjunction with qualitative methods such as quantitative comparative analysis (QCA), which has been proved useful in policy research.^28^

The above theoretical and methodological suggestions do not detract from the important contribution of LSL to expose the consequences of the TPP for the public health of American and Asian countries. The complement of a political analysis with realist methods might reveal an additional understanding of trade agreements to inform actions in health and social policy. In that sense, the TPP itself is not a finished policy since the two candidates for the presidency of the United States have pledged to stop it, and is quite unpopular among US citizens.^29^ Ultimately, the TPP might be less understood as a policy document (that can be amended with technical changes in its provisions) than as a political process with major implications for global public health.

## Ethical issues

Not applicable.

## Competing interests

Authors declare that they have no competing interests.

## Authors’ contributions

Both authors planned the commentary. CM wrote the first draft. DFM provided comments.

## Authors’ affiliations

^1^Bloomberg Faculty of Nursing University of Toronto, Toronto, ON, Canada. ^2^Dalla Lana School of Public Health, University of Toronto, Toronto, ON, Canada. ^3^School of Medicine, University of Toronto, Toronto, ON, Canada.
